# Role of T1 and T2-mapping in assessing the myocardial interstitium in hypertrophic cardiomyopathy: a cardiovascular magnetic resonance study

**DOI:** 10.1186/1532-429X-15-S1-O32

**Published:** 2013-01-30

**Authors:** Tevfik F Ismail, Andrew Jabbour, Ankur Gulati, Niraj Mistry, Mariana Abdel-Malek, Benjamin Hewins, Rick Wage, Michael Roughton, Pedro F Ferreira, Peter D Gatehouse, David N Firmin, Dudley J Pennell, Peter Kellman, Sanjay K Prasad

**Affiliations:** 1 CMR Unit & NHLI Imperial College London, Royal Brompton Hospital & NHLI Imperial College London, London, UK; 2 Laboratory of Cardiac Energetics, National Institutes of Health, Bethesda, MD, USA

## Background

Fibrosis is thought to play an important role in the pathogenesis of the adverse sequelae of hypertrophic cardiomyopathy (HCM). Late gadolinium enhancement (LGE) cardiovascular magnetic resonance detects replacement fibrosis but cannot presently detect interstitial fibrosis. The latter is thought to be a very early feature of HCM and can be triggered by inflammation, which can also expand the size of the myocardial interstitium. We sought to determine whether extracellular volume (ECV) mapping by T1-relaxometry in concert with T2-mapping can identify interstitial changes in HCM and whether these were related to myocardial edema.

## Methods

Twenty HCM patients (mean±SD age: 59.9±9.1 years, 15 male) and 13 healthy volunteers (46.1±11.5 years, 7 male) were studied. Left ventricular T1 and T2 maps were acquired at the base, mid-ventricle and apex in patients and at the mid-ventricle in controls using a modified Look-Locker inversion recovery sequence and a T2-prepared steady-state free precession mapping sequence. Post-contrast T1 maps were acquired a minimum of 15 min after 0.1 mmol/kg gadolinium together with conventional LGE images. After motion correction, pre- and post-contrast T1 parameter maps were co-registered to generate ECV parameter maps. Parameter maps and LGE images were viewed side-by-side and regions of interest (ROI) were drawn in areas with and without LGE. Data were analysed using a linear mixed effects model to account for clustering of ROI within patients.

## Results

**Figure 1:**
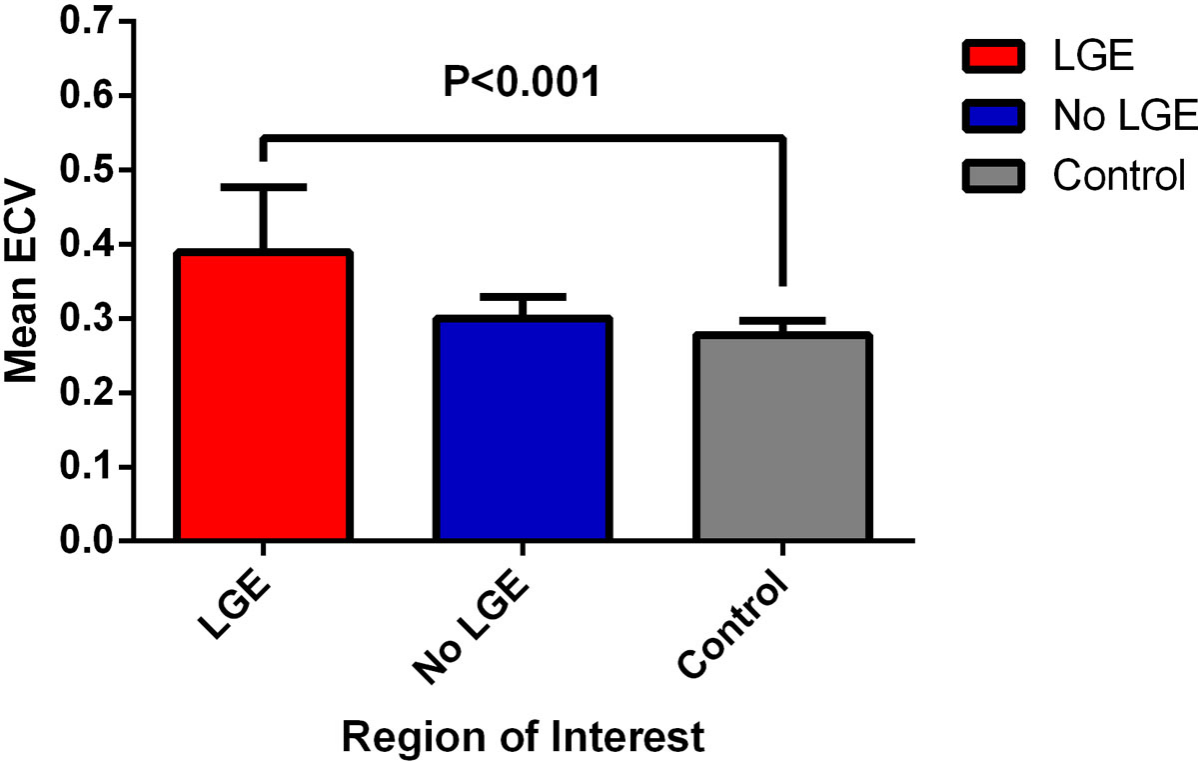
Extracellular Volume Fraction (ECV) in areas with and without Late Gadolinium Enhancement (LGE) in patients with Hypertrophic Cardiomyopathy versus healthy controls.

ROI analysis revealed a significantly higher ECV in regions with LGE relative to controls (0.389±0.087 versus 0.278±0.019, P<0.001)[Figure 1]. However, when compared with controls, there was no significant difference in the ECV of regions from HCM patients that were free of LGE (0.300±0.029 versus 0.278±0.019, P=0.199). T2-times in regions with LGE were similar to controls (55.4±2.6 ms versus 54.9±1.8 ms, P=0.337, respectively) as were the T2 times obtained for regions free of LGE (55.7±2.7 ms versus 54.9±1.8 ms, P=0.309).

## Conclusions

ECV mapping in HCM detects replacement fibrosis, but was unable to detect interstitial fibrosis. There was no evidence of edema in areas of LGE suggesting that this was not a major contributor to ECV expansion in our HCM cohort.

## Funding

This work is supported by the NIHR Cardiovascular Biomedical Research Unit at Royal Brompton and Harefield NHS Foundation Trust, and Imperial College, London, UK. Dr Ismail is supported by the British Heart Foundation. Dr Kellman is supported by the National Institutes of Health, USA.

**Table 1:** 

Characteristic - Mean±SD	Controls (n=13)	Hypertrophic Cardiomyopathy (n=20)	P value
Male - n(%)	7 (53.9)	15 (75.0)	0.208
Age - years	46.1±11.5	59.9±9.1	<0.001
Hematocrit	0.41±0.04	0.41±0.03	0.869
LV wall thickness - mm	8.4±1.5	20.7±3.8	<0.001
LV-EDV index - ml/m^2^	78.9±12.0	69.7±12.9	0.047
LV-ESV index - ml/m^2^	25.0±7.1	19.3±6.8	0.027
LV ejection fraction - %	68.8±6.1	72.8±6.9	0.101
LV Mass Index - g/m^2^	57.4±11.6	93.8±26.0	<0.001

LV=Left Ventricular; LV-EDV=Left Ventricular End-diastolic volume; LV-ESV=Left Ventricular End-systolic volume.

